# Unexpected Discovery of Small Bowel Angiosarcoma Amidst the Management of Severe Polytrauma: A Case Report

**DOI:** 10.7759/cureus.79324

**Published:** 2025-02-19

**Authors:** Mariah Janowski, Mitchell Rentschler, Indu Srinivasan, John Brown, Paul Del Prado

**Affiliations:** 1 Department of Surgery, Creighton University School of Medicine, Phoenix, USA; 2 Department of Clinical Research, Midwestern University Arizona College of Osteopathic Medicine, Glendale, USA; 3 Interventional Gastroenterologist, Valleywise Health Medical Center, Phoenix, USA; 4 Department of Gastroenterology, Creighton University School of Medicine, Phoenix, USA; 5 Pathology, Valleywise Health Medical Center, Phoenix, USA; 6 General Surgery, Valleywise Health Medical Center, Phoenix, USA

**Keywords:** angiosarcoma, case report, diagnosis, gastrointestinal bleeding, small intestine

## Abstract

Angiosarcoma is a rare, soft tissue tumor arising from lymphatic and vascular endothelial cells with high malignancy potential. While commonly observed in cutaneous and subcutaneous tissues, gastrointestinal subtypes are exceedingly rare. A 47-year-old male initially presented for the management of extensive burn injuries sustained in a motor vehicle collision. Throughout an extended hospital stay, he developed melanotic stools and progressive microcytic anemia. Following institutional burn protocol, the patient received multiple blood transfusions. Physical examination revealed a toxic appearance with abdominal distention. A push enteroscopy identified an ulcerated mass in the proximal jejunum. Surgical resection of the mass was performed, and pathological analysis confirmed the diagnosis of gastrointestinal angiosarcoma. The study concludes that patients presenting with unidentifiable melena and anemia after imaging and routine scopes should be considered for having a gastrointestinal lesion, including a gastrointestinal angiosarcoma, despite its rarity. Early recognition of such conditions may facilitate timely diagnosis and intervention, potentially improving survival outcomes. Furthermore, in the complexity of this case in relation to other existing medical conditions and traumatic injuries, weighing risk and benefit to tailor treatment including utilization of chemotherapy and radiation should be individualized.

## Introduction

Angiosarcoma is a highly aggressive and rare malignant soft tissue tumor first described by Schmidt in 1887 in the breast [1-3]. Representing 1-2% of all sarcomas, angiosarcoma carries a poor prognosis, with overall survival ranging from six to 16 months [1,2,4]. The poor prognosis is due to angiosarcomas' ability to rapidly spread hematogenously after insidious growth leading to metastasis before diagnosis; moreover, angiosarcomas have a high rate of local recurrence following treatment [1,2,4]. Standard treatment involves a multi-modal approach including surgical resection, radiation therapy, and chemotherapy [1,2,5,6]. While angiosarcomas originate from lymphatic and vascular endothelial cells, its pathogenesis remains unclear. However, associated risk factors include prior radiation, environmental carcinogens, and chronic lymphedema [1,2,7,8]. Diagnosis is challenging due to the nonspecific nature of imaging findings and requires histological identification revealing spindled, polygonal, and epithelioid cells with vascular and endothelial markers on immunohistochemistry including CD31, CD34, and vascular endothelial growth factor (VEGF) [2,4]. Angiosarcomas can occur anywhere in the body but are typically found in superficial tissues, including the head and neck, breast, and extremities [1,2,9]. Gastrointestinal angiosarcoma is particularly rare and often presents with nonspecific symptoms such as nausea, vomiting, abdominal pain, melena, and fatigue [5,10,11]. This case report will discuss the complexity of diagnosing a jejunal angiosarcoma and the challenges of managing a 47-year-old male with severe polytrauma following a motor vehicle collision.

## Case presentation

A 47-year-old male, critically injured in a motor vehicle collision, was admitted for extensive burn injuries involving approximately 33% of the total body surface area (TBSA) as full-thickness burns. Following initial trauma care, the patient underwent an exploratory laparotomy and multiple percutaneous fracture repairs from days 1 to 3. From day 3 to day 74, the patient was managed in the burn intensive care unit, where he underwent 12 tissue expansion surgeries, three surgical excision debridements, three biodegradable temporizing matrix procedures, and four skin grafts across his body.

On hospital day 78, the patient developed progressive anemia requiring repeated transfusions, a declining trend in hemoglobin levels, and new-onset melanotic stools, prompting a gastroenterology consultation (Table 1).

**Table 1:** Complete blood count trends Blood count trends from day 78 at the start of melanotic stools to day 87 the day of general surgery consultation. WBC: white blood cell; HGB: hemoglobin; HCT: hematocrit; PLT: platelet; µL: microliters; g: grams; H: high; L: low

Day	Day 78	Day 78	Day 78	Day 79	Day 87	Day 87
Time	0408	1149	1932	0253	0409	1021
WBC (cells/µL)	9.9	--	13.1 (H)	10.9	12.2 (H)	11.5 (H)
HGB (g/dL)	7.3 (L)	7.4 (L)	7.0 (L)	8.5 (L)	8.3 (L)	7.9 (L)
HCT (%)	21.4 (L)	22.5 (L)	20.8 (L)	24. 9 (L)	24.1 (L)	22.9 (L)
PLT (cells/µL)	193	--	181	176	201	208

**Figure 1:**
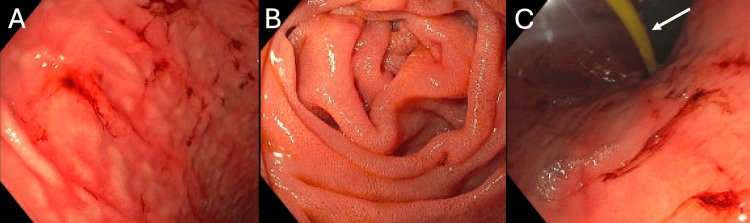
Day 79 esophagogastroduodenoscopy Imaging of the gastric fundus (A) revealed minor spots of blood and mild gastropathy, without evidence of erosions, ulcers, or lesions. Imaging of the first (B) and second (C) parts of the duodenum showed normal mucosa, with no erosions, and the presence of a nasoduodenal tube (arrow).

**Figure 2:**
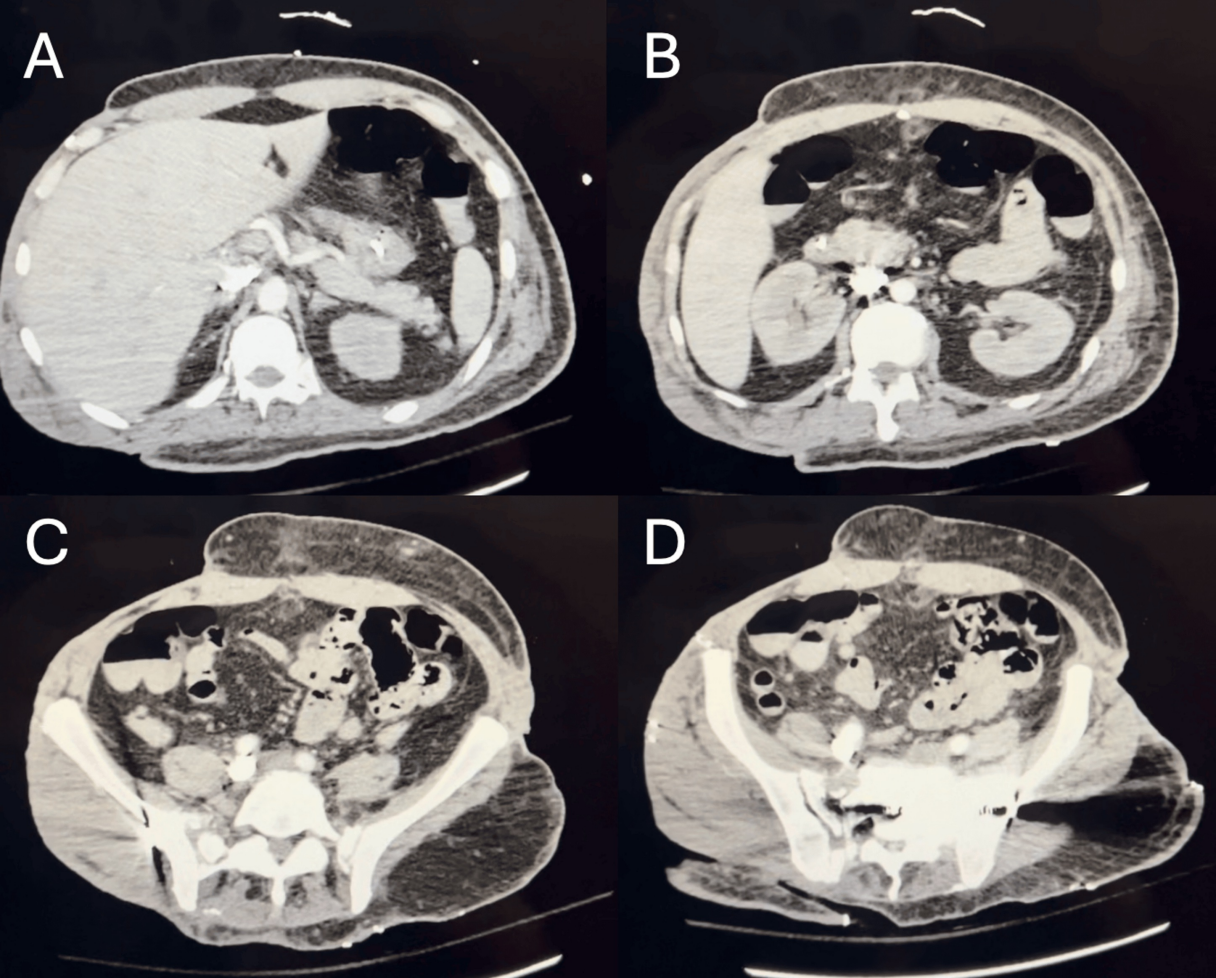
Day 81 computed tomography angiography Imaging of the celiac (A) and superior mesenteric (B) arteries, as well as the gastrointestinal tract (C and D), showed no evidence of active bleeding.

The patient appeared ill and toxic-appearing, with 720 milliliters (mL) of dark tarry stool collected from a fecal management system (FMS). On day 79, an esophagogastroduodenoscopy (EGD) was performed, which revealed a normal esophagus, mild gastropathy with localized spots of blood but no erosions or ulcers, and a normal duodenum without evidence of erosions (Figure 1). On day 81, a computed tomography (CT) angiography was conducted, showing no abnormalities or signs of active gastrointestinal bleeding (Figure 2).

**Figure 3:**
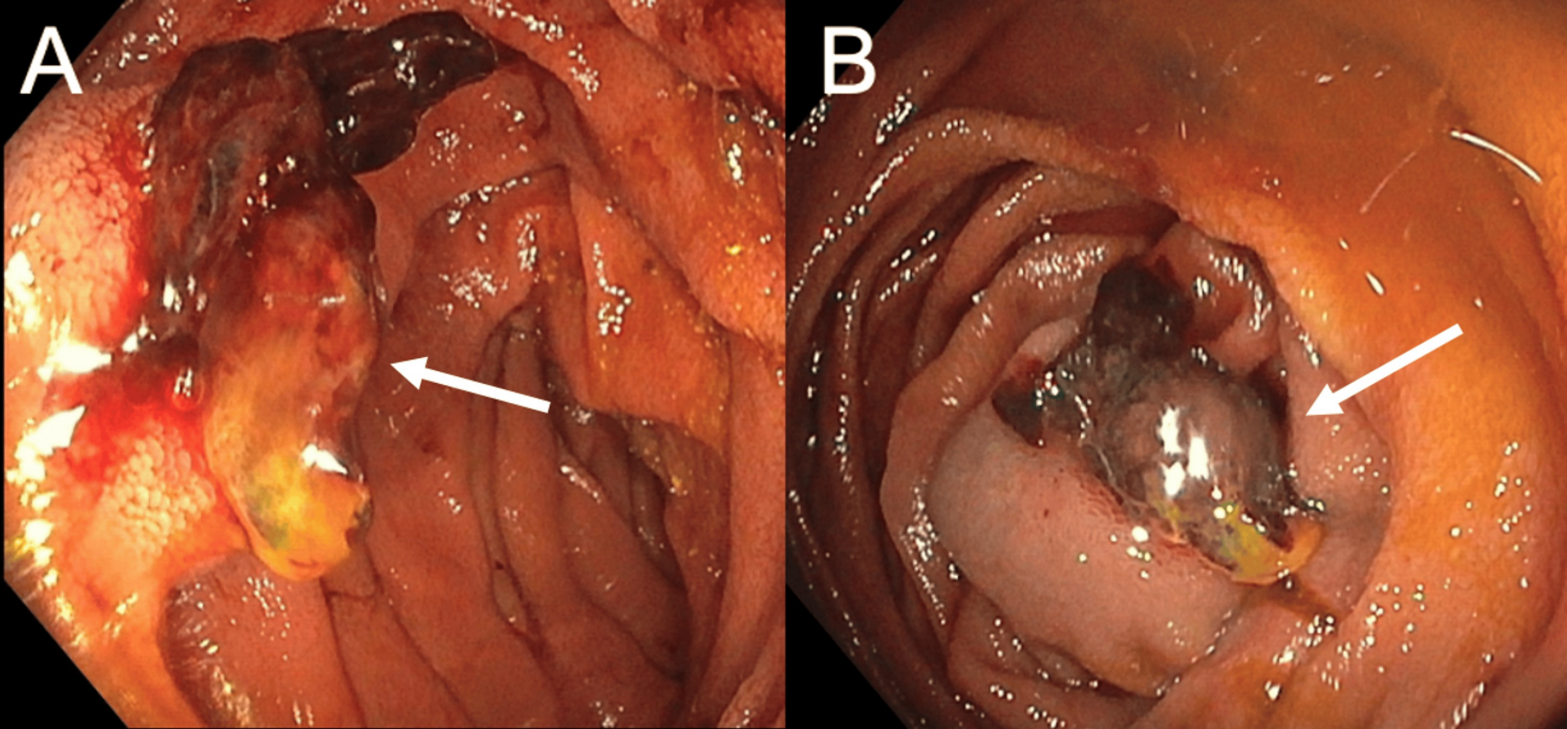
Day 86 push enteroscopy Imaging (A and B) from the push enteroscopy revealed a 1.5 cm raised ulcerated lesion in the proximal jejunum. Pathological analysis following resection diagnosed the lesion as an angiosarcoma.

A colonoscopy on day 84 demonstrated unremarkable terminal ileum and colonic mucosa, although a clean-based ulcer was noted on the posterior wall of the anal canal, likely resulting from the FMS. No images were taken during the colonoscopy. The original source of the bleeding remained undetermined, as the tarry stools had occurred before FMS insertion. Finally, on day 86, a push enteroscopy identified a 1.5 cm raised, ulcerated lesion in the jejunum (Figure 3).

General surgery was consulted on day 87. The patient’s vitals included a blood pressure of 132/61 millimeters of mercury (mmHg), pulse of 121 beats per minute, temperature of 37.3 °Celsius (C), respiratory rate of 40 breaths per minute, and oxygen saturation at 100%. A complete blood count was collected with results shown in Table 1. Due to extensive burn injuries, a thorough physical examination was not feasible. On day 89, the patient underwent an enterectomy with anastomosis and insertion of feeding jejunostomy, a 10 millimeter (mm) mass was located 57 cm from the ligament of Treitz and was resected with 5 cm proximal and distal margins. A side-to-side, anti-peristaltic stapled anastomosis was performed, and a feeding jejunostomy was placed 20 cm distal to the anastomosis.

**Figure 4:**
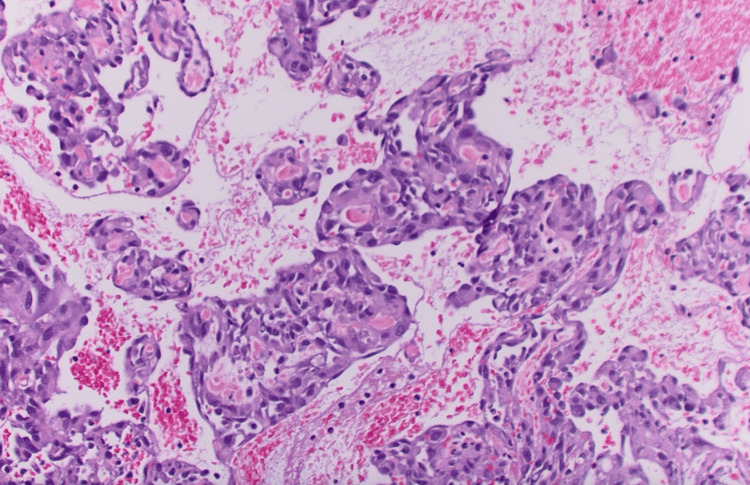
Composite hematoxylin and eosin (H&E)-stained tumor Composite H&E-stained sections of tumor, revealed interrupted, atypical vascular and swollen endothelial cells lining open vascular channels at the base of the lesion. The lesion extended into the submucosa and partially affected the muscularis propria, with few mitotic figures observed.

Pathological analysis identified the lesion as a polypoid mass with extensive surface ulceration and hemorrhage. The base of the lesion demonstrated disrupted atypical vascular and plump endothelial cells outlining open vascular channels (Figure 4).

**Figure 5:**
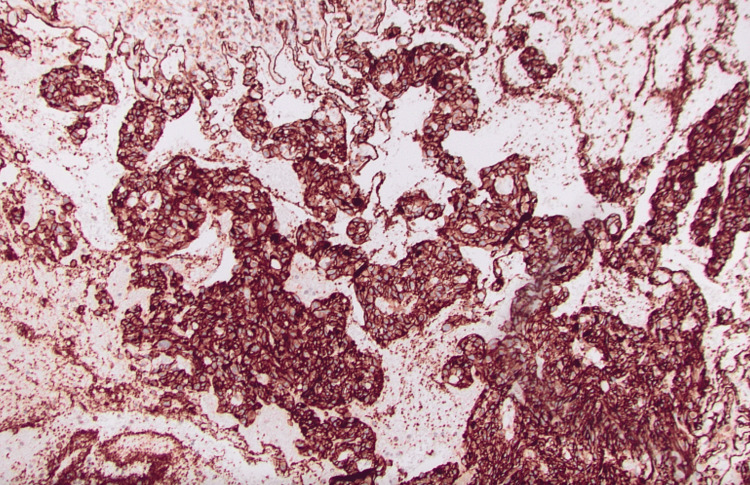
CD 31 immunohistochemistry staining Immunohistochemistry staining for CD 31 demonstrated strong positive staining of the neoplastic endothelial cells.

The lesion extended into the submucosa and partially involved the muscularis propria and rare mitoses were noted (Figure 2). Immunohistochemistry revealed strong vascular endothelium reactivity with a cluster of differentiation (CD) 31 and negativity for CD 117 (Figure 5).

Surgical margins were clear of the tumor, and one benign lymph node negative for neoplasm was identified. Following the diagnosis of gastrointestinal angiosarcoma, a thoracentesis was conducted as per oncology recommendations; pleural fluid analysis was negative for malignancy.

Due to the patient’s critical condition and extensive hospital stay, chemotherapy and radiation were deferred due to associated risks. The clear surgical margins and negative pleural fluid analysis suggested no immediate evidence of metastasis.

The patient's hospital stay was marked by *Acinetobacter pneumonia*, which was initially treated with amphotericin and later switched to oral isavuconazonium sulfate (Cresemba). He eventually underwent a tracheostomy, which was later decannulated. His diet was modified to a mechanical soft diet with additional nighttime tube feeds. After four months, he was discharged to a facility and returned home four weeks later.

## Discussion

In 1887, Schmidt first described angiosarcoma in the breast, followed by subsequent identifications of deep soft tissue angiosarcoma in 1976 [3,10]. Angiosarcomas preferentially occur in the head, neck, breast, and cutaneous locations but can present anywhere in the body [1,2,9,12]. The incidence of angiosarcoma in the United States is approximately 3.3 cases per 1,000,000 people [13]. However, the incidence significantly decreases for gastrointestinal angiosarcoma subtypes, with less than a hundred documented case reports and small sample size case series globally [5,6,11,14]. Although angiosarcoma often arises spontaneously, several risk factors have been identified including age, sex, prior radiation, chronic lymphedema, exposure to toxins, and genetic mutations [1,3,5,11-13]. Notable carcinogens include vinyl chloride and thorium dioxide, while NF-1 (neurofibromatosis), IDH1 (maffucci syndrome), and breast cancer gene (BRCA) 1 and 2 are associated genetic mutations [1,2,11,12].

Angiosarcoma is classified as a rare, highly aggressive malignant tumor, representing approximately 1-2% of all sarcoma diagnoses [1,2,4,13,14]. Furthermore, angiosarcomas exhibit a high rate of local recurrence and are typically metastatic at the time of diagnosis [4]. Due to these factors, angiosarcomas have a poor prognosis, with survival ranging from six to 16 months [1,2,4,14].

Angiosarcomas originate from lymphatic or vascular endothelial cells, but the pathogenesis remains unclear [1,2,4,15]. Current theories suggest that malignant transformation occurs in a favorable microenvironment involving a complex interaction of established risk factors, such as prior radiation, chronic inflammation, tissue remodeling, and genetic factors [15]. Once established, the inherently aggressive nature of angiosarcoma leads to hematogenous spread, with the lungs and liver being the most common sites of metastasis [3,4].

Diagnosis is often delayed due to vague presenting symptoms and challenges in imaging identification [4,5,12,15]. Gastrointestinal angiosarcoma typically presents with nonspecific melena, anemia, abdominal pain, nausea, vomiting, and fatigue [4,5]. These nonspecific symptoms tend to decrease the suspicion of intestinal angiosarcoma due to its rarity and the prevalence of more common diagnoses [5]. Radiological characteristics of gastrointestinal tract angiosarcoma are not well established; however, case reports suggest that CT and magnetic resonance imaging (MRI) may reveal thickened intestinal walls with varying degrees of enhancement in the arterial phase [4,6,11]. Contrast-enhanced CT imaging of angiosarcomas in other locations may provide some reference with CT imaging of breast and soft tissue angiosarcomas typically showing irregular, uncalcified, and hypoechoic masses [4]. Given the nonspecific symptoms and unclear imaging, colonoscopy and EGD are typically recommended, revealing a grayish-red ulcerated lesion [6,11]. According to the American College of Gastroenterology (ACG) guidelines for small bowel bleeding, if colonoscopy and EGD are unremarkable, a push enteroscopy and video capsule endoscopy are indicated [16]. In this case, the guidelines were followed, and it is unlikely that the angiosarcoma would have been identified sooner. Histological and immunochemical biopsies remain the gold standard for diagnosis [2]. Histologically, angiosarcoma is characterized by polygonal, rounded, spindled endothelial cells with immunochemical analysis positive for CD31, CD34, von Willebrand factor, and vascular endothelial growth factor (VEGF) [2,4,12].

Treatment for angiosarcoma is based on prognosis, but the aggressiveness, complexity, and lack of specific prognostic factors make it difficult to establish a definitive prognosis, which is typically poor [12,17,18]. Studies investigating prognostic factors have indicated that prolonged survival may be associated with radiation, chemotherapy, and surgical resection [12,18]. Yuan-Yuan et al. have developed a nomogram with good predictive ability for angiosarcoma; however, these studies focus on angiosarcoma as a whole rather than the gastrointestinal subtypes [17]. Current recommendations for the treatment of localized angiosarcomas include surgical resection with wide margins, followed by radiation and chemotherapy, which have been shown to increase survival time from six to 16 months to 24 months compared to surgical resection alone; however, no standard protocol has been established [1,2,5,6,10-12,14]. Adjuvant radiation and chemotherapy are associated with improved survival due to a high rate of reoccurrence, with the most effective and studied chemotherapeutic agents being anthracyclines, taxanes, VEGF inhibitors, and tyrosine kinase inhibitors [1,2,5,6,10-12,14,18,19]. Adjuvant radiation is recommended for all angiosarcoma treatments, especially following postoperative surgical resection of localized tumors, due to the high rate of local recurrence [1,2,14]. The EORTC clinical trials demonstrated that doxorubicin and taxanes reduce local metastasis and are considered first-line treatments for all angiosarcomas, although the overall survival time remains between 6 and 16 months [1,2,5,12,18,19]. The use of VEGF inhibitors and tyrosine kinase inhibitors has shown mixed results in extending overall survival time and is currently reserved for second- and third-line treatments [1,2,5,18,19]. Although these guidelines exist, due to the complexity of this case, including the presence of other medical conditions and traumatic injuries, the decision to use treatments like chemotherapy and radiation should be personalized based on a careful assessment of risk and benefits.

## Conclusions

Angiosarcoma is a highly malignant and rare soft tissue tumor originating from lymphatic and vascular endothelial cells. This tumor often presents with vague symptoms, and metastasis is commonly present at the time of diagnosis. Angiosarcoma diagnosis is difficult on imaging so the gold standard of diagnosis remains histological and immunohistochemical analysis. Primary treatment modalities include surgical resection, chemotherapy, and radiation; however, the prognosis remains generally poor. Clinicians should consider gastrointestinal lesions, including angiosarcomas, in patients presenting with melena, anemia, and a persistent decrease in hemoglobin, particularly in those with relevant risk factors, to facilitate prompt treatment and potentially improve patient survival. While guidelines provide a framework for treatment, complex cases involving other medical conditions and traumatic injuries require that the treatment such as chemotherapy and radiation be individualized, with careful consideration of risks and benefits as in this case where the patient only underwent surgical resection. The patient eventually returned home and was fortunate to detect the angiosarcoma while it was still localized; however, he will require recurrent follow-up due to the high likelihood of reoccurrence. Further studies are needed to investigate treatment options and prognostic factors to better guide the management of patients with angiosarcoma, especially for the rare gastrointestinal subtypes and with complicating medical factors.
